# Application of Computational Lower Extremity Model to Investigate Different Muscle Activities and Joint Force Patterns in Knee Osteoarthritis Patients during Walking

**DOI:** 10.1155/2013/314280

**Published:** 2013-11-04

**Authors:** Kyung Wook Nha, Ariunzaya Dorj, Jun Feng, Jun Ho Shin, Jong In Kim, Jae Ho Kwon, Kyungsoo Kim, Yoon Hyuk Kim

**Affiliations:** ^1^Department of Orthopaedic Surgery, Inje University, Ilsan Paik Hospital, Ilsan 441-706, Republic of Korea; ^2^Department of Mechanical Engineering, Kyung Hee University, Yongin 446-701, Republic of Korea; ^3^Department of Applied Mathematics, Kyung Hee University, Yongin 446-701, Republic of Korea

## Abstract

Many experimental and computational studies have reported that osteoarthritis in the knee joint affects knee biomechanics, including joint kinematics, joint contact forces, and muscle activities, due to functional restriction and disability. In this study, differences in muscle activities and joint force patterns between knee osteoarthritis (OA) patients and normal subjects during walking were investigated using the inverse dynamic analysis with a lower extremity musculoskeletal model. Extensor/flexor muscle activations and torque ratios and the joint contact forces were compared between the OA and normal groups. The OA patients had higher extensor muscle forces and lateral component of the knee joint force than normal subjects as well as force and torque ratios of extensor and flexor muscles, while the other parameters had little differences. The results explained that OA patients increased the level of antagonistic cocontraction and the adduction moment on the knee joint. The presented findings and technologies provide insight into biomechanical changes in OA patients and can also be used to evaluate the postoperative functional outcomes of the OA treatments.

## 1. Introduction

Osteoarthritis (OA) is regarded as a degenerative joint disease that results from loss of balance between the biological resistance of the joint and mechanical stress applied to the joint, and it is defined as conditions that lead to joint symptoms and signs associated with defects in the integrity of articular cartilage, in addition to related changes in the underlying bone at the joint margins [1, 2]. The knee is one of the most common joints afflicted by OA with aging: about 33% of people older than 65 have OA in their knee joints [2]. Many experimental and computational studies have reported that OA in the knee joint affects knee biomechanics, including joint kinematics, joint contact forces, and muscle activities, due to functional restriction and disability [3, 4]. 

Several experimental studies have measured periarticular knee muscle activation of OA patients during walking using electromyography (EMG) systems [5–8] and knee joint contact forces using instrumented knee implants [9–12]. Even though direct measures would be more realistic, direct measures are invasive and require special devices, for example, instrumented implants, fine-wires, or indwelling electrodes. Although surface EMG is not invasive, it generally measures the activity of superficial muscles. In the lower extremity, surface EMG can be used to measure the activation of medial and lateral hamstrings, medial and lateral vastus, and rectus femoris, but it is difficult to estimate activation of the vastus intermedius, semimembranosus, and semitendinosus, separately [13].

The most common noninvasive method is computational analysis based on inverse dynamic analysis that uses a virtual model of the knee joint. This type of analysis allows prediction of muscle forces, mechanical stresses, and joint forces that are impossible or very difficult to measure *in vivo*. Several studies have investigated abnormalities in individuals with OA in the knee joint from the perspectives of movement kinematics, joint loads, and joint stability [14–19]. It has been shown that OA is often accompanied by increased knee laxity and stiffness, reduced flexion angles, and increased adduction or decreased flexion moments. Knee joint constraint forces and muscle force predictions were analyzed in OA patients by inverse dynamic methods. Pain relief efficiency to knee contact force in subjects with knee OA was significantly different from that of healthy controls [20]. However, differences in muscle forces and joint contact forces between OA patients and healthy people have not been investigated in detail in the previous studies.

In this study, differences in muscle activity and joint force patterns between knee OA patients and normal subjects during walking were investigated using the motion capture data and inverse dynamic analysis with a lower extremity model. Extensor/flexor muscle activation ratio and torque ratio were calculated to compare muscle activities, and three-dimensional (3D) components (compressive, medial-lateral, and anterior-posterior) of the joint contact force were analyzed.

## 2. Methods

### 2.1. Subjects

Two groups of subjects participated in this study after providing informed consent: 11 OA patients (age, 53.8 ± 5.5 yrs; height, 160.0 ± 8.0 cm; weight, 73.1 ± 13.4 kg) who had medial knee OA diagnosed according to the criteria defined by the American College of Rheumatology (radiographic analyses or clinical tests) [1] and 10 asymptomatic participants (age, 26.7 ± 1.7 yrs; height, 164.0 ± 5.4 cm; weight, 58.5 ± 10.5 kg) with no clinical diagnosis of OA, rheumatoid arthritis, or history of knee trauma or pain. Participants were able to walk at least 40 m independently, and potential participants were excluded if they had uncontrolled systemic disease or preexisting neurological or other orthopaedic conditions affecting their ability to walk.

### 2.2. Experiments

**Figure 1:**
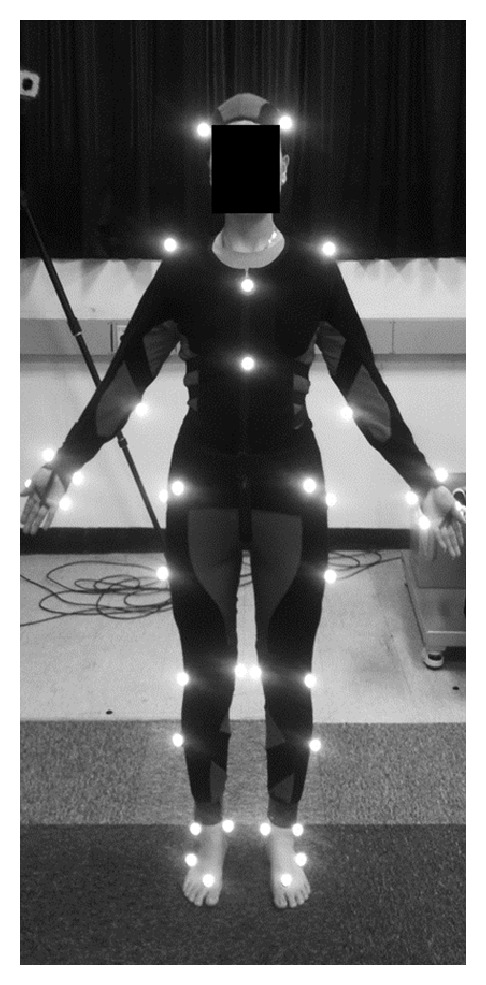
Positions of 37 retroreflective markers for motion capture during gait cycles.

A 3D motion analysis system (Hawk Digital Real Time System, Motion Analysis System, Santa Rosa, CA, USA) with 10 cameras operating at a sampling rate of 100 Hz was used to record motion capture data. In addition, four 1000 Hz force plates (MP4060, Bertec Corporation, Columbus, OH, USA) were used to measure ground reaction forces and identify gait cycle events. Thirty-seven retroreflective markers were attached to the lower extremities (bilateral anterior superior iliac spines, posterior superior iliac spines, lateral/medial femoral condyles, lateral/medial malleolus, forefeet, and heels) and the upper body (sternum, processus xiphoideus, C7 vertebra, T10 vertebra, vertex, bilateral front of head, right/left acromion, medial/lateral epicondyles, radial/ulnar styloid, and second and fifth metacarpals), based on previous positions reported in the literature [22, 23] (Figure 1). Subjects walked at a self-selected comfortable walking speed along a 6 m walkway after multiple trials to ensure that they were able to walk comfortably with consistent velocity. Data from five trials were averaged; these averaged values were used in the analysis.

### 2.3. Dynamic Model

**Figure 2:**
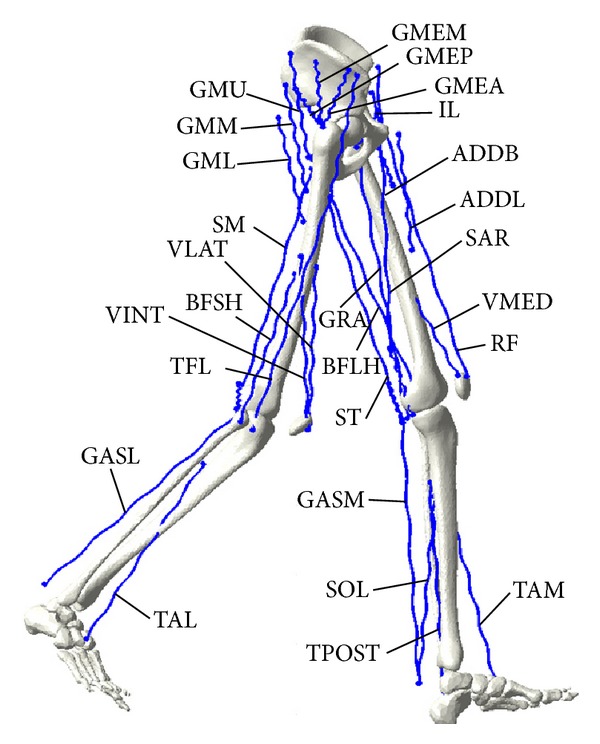
Muscles in the lower extremity model. Abbreviations are as follows [21]: vastus intermedius (VINT); vastus lateralis (VLAT); vastus medialis (VMED); rectus femoris (RF); semimembranosus (SM); semitendinosus (ST); biceps femoris long head (BFLH); biceps femoris short head (BFSH); gastrocnemius medial (GASM); gastrocnemius lateral (GASL); tensor fasciae latae (TFL); soleus (SOL); tibialis posterior (TPOST); tibialis anterior lateral (TAL); tibialis anterior medial (TAM); gluteus maximus upper (GMU); gluteus maximus middle (GMM); gluteus maximus lower (GML); gluteus medius posterior (GMEP); gluteus medius middle (GMEM); gluteus medius anterior (GMEA); adductor brevis (ADDB); adductor longus (ADDL); iliacus (IL); sartorius (SAR); and gracilis (GRA).

 A dynamic model of the lower extremities was constructed to calculate kinematics and kinetics such as joint angle, angular velocity, angular acceleration, moment, joint constraint forces, and muscle forces during the gait cycle. The model consisted of 6 segments and 18 degrees-of-freedom linkages for the hip, knee, and ankle joints (Figure 2). Mass, center of gravity, and moment of inertia of each body segment were determined by scaling according to the subject's body weight and segmental lengths obtained from the markers based on [24]. Local coordinate systems for each body segment were set based on the recommendations of the International Society of Biomechanics [22, 23]. Joint centers of the hip, knee, and ankle were defined by marker positions specified in [25]. Twenty-six pairs of lower extremity muscles were then considered, and the origin and insertion sites of each muscle were extracted from the literature [21] (Figure 2).

### 2.4. Inverse Dynamics

Knee joint kinematic information, such as angular velocity and acceleration of the *i*th segment, was obtained from motion capture data by using the finite difference technique. The net joint force and moment acting on the *i*th body segment were then calculated starting from the distal segment with ground reaction force and moment [26, 27] by using the following equilibrium equations:

(1) $$\begin{array}{c} \vec{F}{}_{i}^{e}=m_{i}\left({\vec{a}}_{i}-\vec{g}\right)-\vec{F}{}_{i-1}^{e},\\ \vec{M}{}_{i}^{e}=I_{i}{\vec{\alpha }}_{i}+{\vec{\omega }}_{i}\times \left(I_{i}{\vec{\omega }}_{i}\right)-{\vec{l}}_{i}\times \vec{F}{}_{i}^{e}-{\vec{l}}_{i-1}\times \vec{F}{}_{i-1}^{e}-\vec{M}{}_{i-1}^{e}, \end{array}$$

where *m*_*i*_ is the *i*th segmental mass, ${\vec{a}}_{i}$ is the translational acceleration vector of the *i*th segment's center of gravity, $\vec{g}$ is the gravitational vector, *I*_*i*_ is the moment of inertia around the center of gravity of the *i*th segment, ${\vec{\omega }}_{i}$ and ${\vec{\alpha }}_{i}$ are the angular velocity and acceleration vector of the *i*th segment, ${\vec{l}}_{i}$ is the distance from the segmental center of gravity of the *i*th segment to the distal joint center, and $\vec{F}{}_{i-1}^{e}$ and $\vec{M}{}_{i-1}^{e}$ are the jet joint force and moment acting on the distal segment, called the (*i* − 1)th segment.

The corresponding joint force and moment equilibrium equations, including muscle forces, were finally formulated for each joint during 100 intervals in one gait cycle as follows:

(2) $$\begin{array}{c} \sum_{i=1}^{26}F_{i}^{M}{\vec{\tau }}_{i}+\vec{F}{}^{j}=\vec{F}{}^{e},\\ \sum_{i=1}^{26}F_{i}^{M}\left({\vec{r}}_{i}\times {\vec{\tau }}_{i}\right)=\vec{M}{}^{e}, \end{array}$$

where *F*_*i*_^*M*^ and ${\vec{\tau }}_{i}$ are the magnitude and unit direction vector of the *i*th muscle force, $\vec{F}{}^{j}$ is the joint constraint force, ${\vec{r}}_{i}$ is the location vector of the *i*th muscle with respect to the joint center, and $\vec{F}{}^{e}$ and $\vec{M}{}^{e}$ are the net joint force and moment, respectively. Muscle forces were calculated using the static optimization technique because of the redundancy of variables [12]. Maximum isometric muscle forces were assumed to be proportional to the physiological cross-sectional area (PCSA), and the maximum values dependent on the muscle group and age were obtained from [21, 28]. The sum of stresses cubed was used as the objective function:

(3) Minimize ∑i=126(FiMAi)3subject to 0≤FiMAi≤σi, i=1,…,26,

where *A*_*i*_ and *σ*_*i*_ are the PCSA and maximum stress of the *i*th muscle, respectively. A customized MATLAB (The MathWorks, Natick, MA, USA) was utilized to solve the above optimization problem. 

 To investigate the knee extensor/flexor ratio, four muscles were selected as knee extensor muscles (VINT, VLAT, VMED, and RF) and eight muscles were chosen as flexor muscles (SM, ST, BFLH, BFSH, GASM, GASL, SAR, and GRA). The knee extensor/flexor muscle activation ratio (EFAR, *A*_*e*_/*A*_*f*_) and knee extensor/flexor torque ratio (EFTR, *T*_*e*_/*T*_*f*_) were defined as follows:

(4) AeAf=(AVINT+AVLAT+AVMED+ARF)×(ASM+AST+ABFLH+ABFSH+AGASM+AGASL+ASAR+AGRA)−1,TeTf=(TVINT+TVLAT+TVMED+TRF)×(TSM+TST+TBFLH+TBFSH+TGASM+TGASL+TSAR+TGRA)−1,

where *A*_*X*_ and *T*_*X*_ represent the muscle force and the torque generated by muscle *X*, respectively.

### 2.5. Data Analysis

The estimated muscle forces and joint reaction forces were averaged for 10 subjects in the asymptomatic group. The Pearson correlation coefficients between the mean values in the asymptotic group and those reported in a previous study [12] at 100 time steps during the gait cycle for the quadriceps (VINT, VLAT, VMED, and RF), hamstring (SM, ST, BFLH, and BFSH), and gastrocnemius (GASM and GASL) as well as the compressive joint force were analyzed to confirm the validity of the present approach. An independent sample *t*-test was then performed to compare the mean values of muscle forces, knee extensor/flexor ratios (EFAR and EFTR) in response to muscle activity, and peak knee joint force components (compressive, medial-lateral, and anterior-posterior) during the gait cycle between 11 OA patients (OA) and 10 normal subjects (asymptomatic), respectively. The IBM SPSS Statistics 20 (SPSS, Chicago, IL, USA) was used for the statistical analysis, and the significant difference was defined as *P* < 0.05. 

## 3. Results

**Figure 3:**
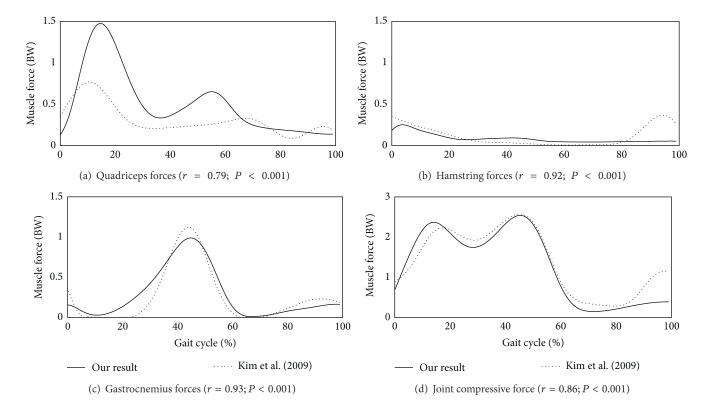
The predicted muscle forces of (a) quadriceps, (b) hamstring, (c) gastrocnemius, and (d) compressive forces and those in [12] during a gait cycle. Our results were averaged for 10 subjects in the asymptotic group, and the forces were normalized to each subject's body weight (BW). The Pearson correlation coefficients and *P* values were also provided.

The averaged values in the asymptomatic group for muscle forces of the quadriceps, hamstring, and gastrocnemius as well as the compressive joint force during a gait cycle were depicted in Figure 3 with those reported in [12]. The Pearson correlation coefficients between our data and the previous data were 0.79 for quadriceps, 0.92 for hamstring, 0.93 for gastrocnemius, and 0.86 for compressive joint force with *P* < 0.001 for all cases.

**Figure 4:**
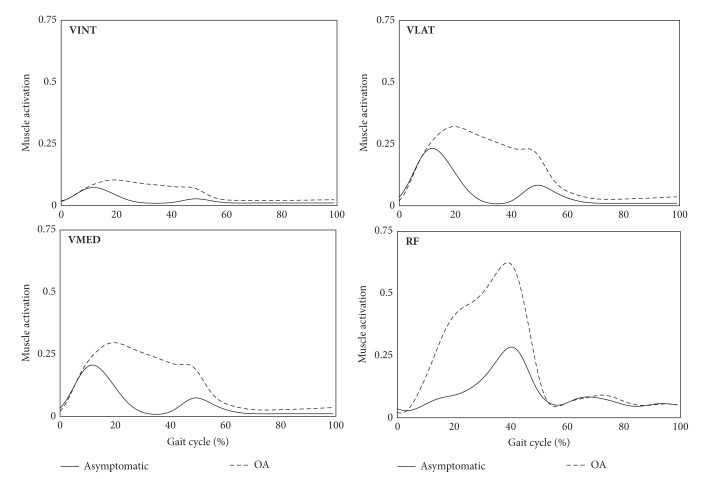
Knee extensor muscle forces for OA patients and asymptomatic subjects during a gait cycle, where the muscle forces were averaged in both groups and scaled to each muscle's maximum isometric force (VINT, vastus intermedius; VLAT, vastus lateralis; VMED, vastus medialis; RF, rectus femoris).

**Figure 5:**
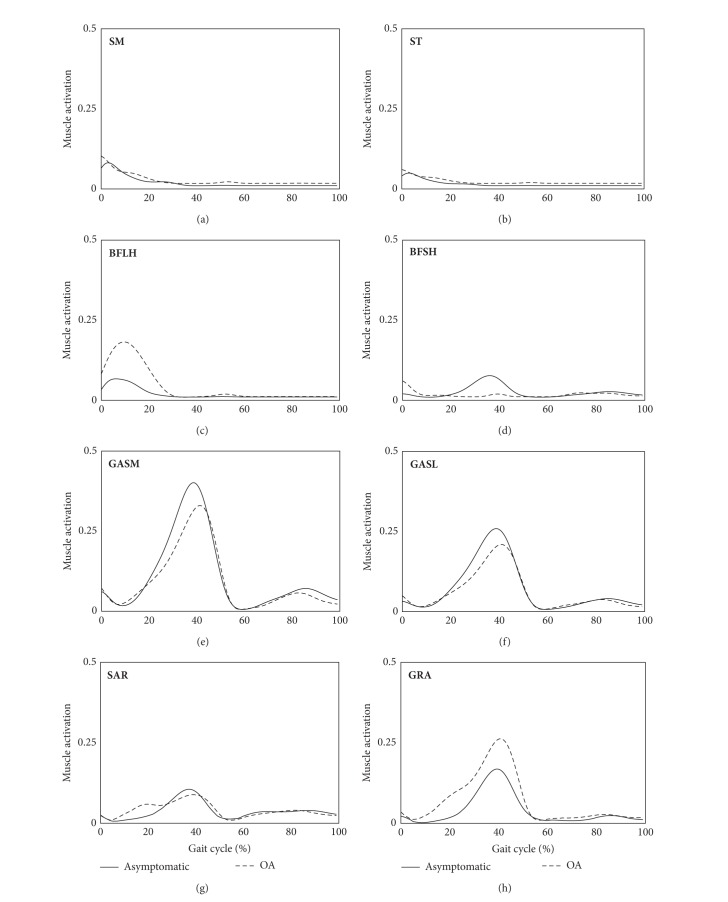
Knee flexor muscle forces for OA patients and asymptomatic subjects during a gait cycle, where the muscle forces were averaged in both groups and scaled to each muscle's maximum isometric force (SM, semimembranosus; ST, semitendinosus; BFLH, biceps femoris long head; BFSH, biceps femoris short head; GASM, gastrocnemius medialis; GASL, gastrocnemius lateralis; SAR, sartorius; GRA, gracilis).

The averaged muscle forces in the knee extensor muscles for both OA and asymptomatic groups are presented in Figure 4, and the *P* values for the *t*-test between the two groups were summarized in Table 1. OA patients produced significantly greater extensor muscle forces than asymptomatic subjects during the overall gait cycle in all extensor muscles. The knee flexor muscles of OA patients exerted higher BFLH muscle forces and lower BFSH muscle forces among the lateral hamstrings, while the forces exerted by the medial hamstrings (SM and ST) were similar (Figure 5 and Table 1). Muscle activation patterns of the gastrocnemius muscles (GASM and GASL) and SAR were similar, while GRA activation was higher in the OA group (Figure 5 and Table 1).

**Figure 6:**
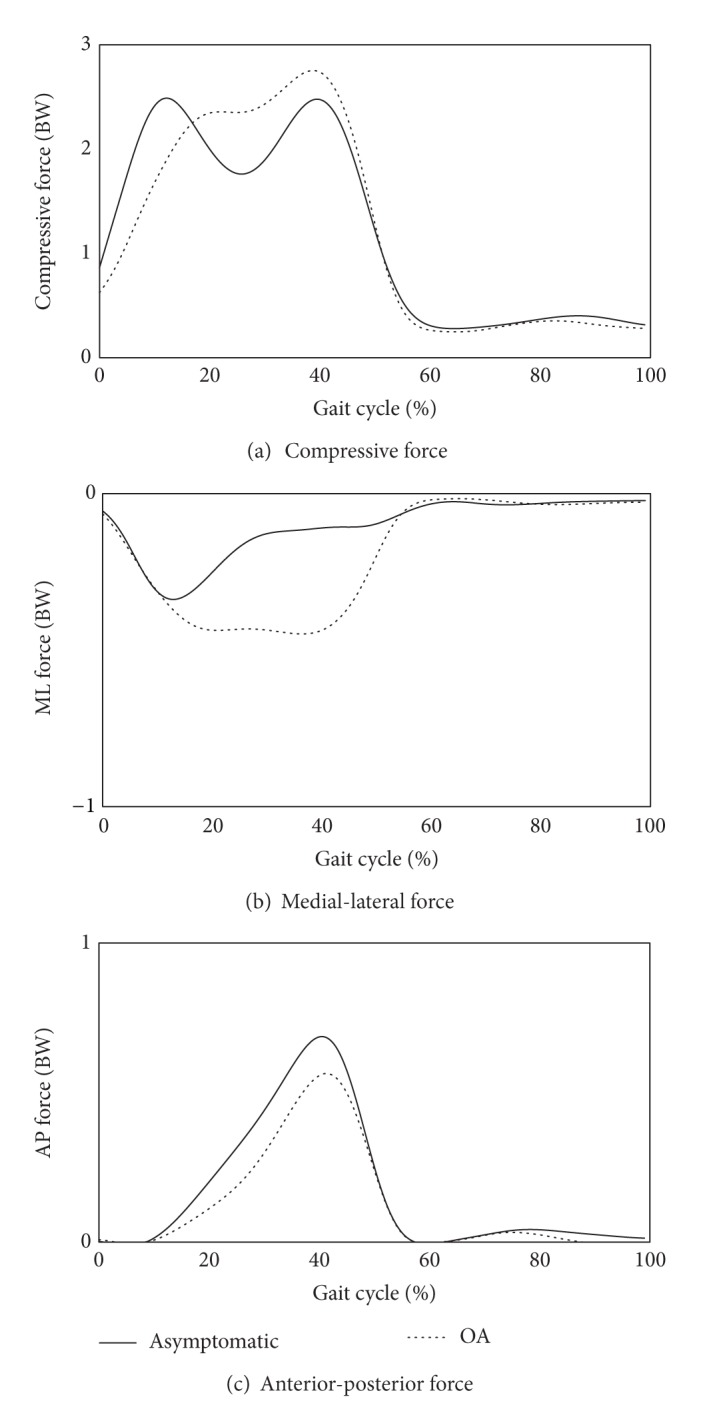
Predicted knee joint forces: (a) compressive forces (positive, tensile force; negative, compressive force), (b) medial-lateral forces (positive, medial; negative, lateral), and (c) anterior-posterior forces (positive, posterior; negative, anterior) of OA patients and asymptomatic subjects, where the joint forces were averaged in both groups and scaled to each subject's body weight (BW).

The averaged 3D components of knee joint forces for both OA and asymptomatic groups are presented in Figure 6. Compressive and medial-lateral (ML) forces calculated for OA patients were higher than those of asymptomatic subjects, although little difference in the maximum magnitude was observed (Table 1). Anterior-posterior (AP) forces were presented similarly between the two groups (Figure 6 and Table 1). 

**Figure 7:**
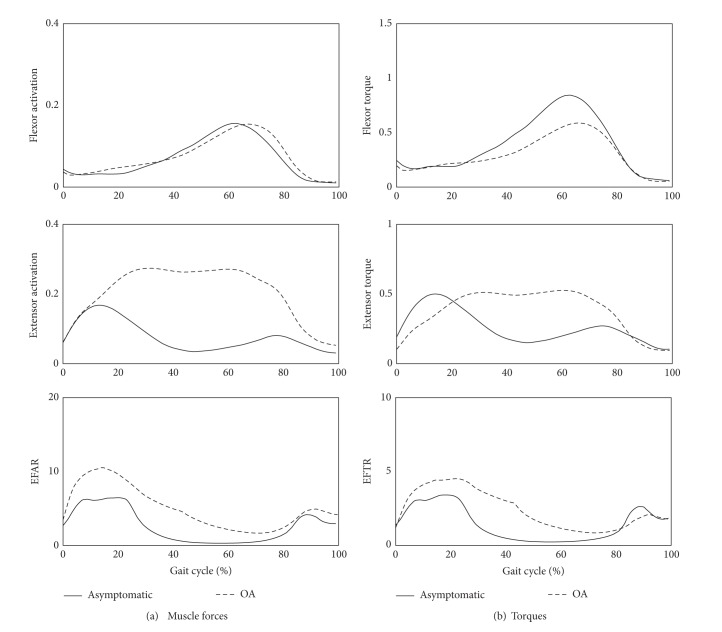
Comparison of (a) muscle forces of flexor and extensor muscle groups and EFAR and (b) torques generated by flexor and extensor muscle groups and EFTR between OA patients and asymptomatic subjects during a gait cycle, where the values were averaged in both groups.

The averaged knee extensor/flexor muscles forces and torques as well as their ratios (EFAR and EFTR) are given in Figure 7. Although there were no large differences in muscle forces or torques for flexor muscles between OA patients and normal controls, OA patients had higher values for extensor muscles (Table 1). Moreover, EFAR and EFTR were substantially higher in OA patients than control subjects for the entire duration of the gait cycle (Table 1).

## 4. Discussion

Muscle forces of the quadriceps (VINT, VLAT, VMED, and RF), hamstring (SM, ST, BFLH, and BFSH), and gastrocnemius (GASM and GASL) as well as the compressive joint force were consistent with those reported in [12], thereby validating our model (Figure 3). Trends in muscle and joint forces were similar, and the Pearson correlation coefficients and the *P* values were enough to show the strong positive correlations. In addition, the peak compressive contact force in the knee joint ranged from 2.5 to 3.1 times body weight (BW) in the asymptomatic group, consistent with the previous experimental results measured *in vivo* (1.6 to 3.5 BW) [9–12].

Previous experimental results for knee extensor and flexor muscle forces support our findings that knee extensor muscle activity was significantly higher in individuals with knee OA than in asymptomatic controls [29]. It was also shown that the muscle activity pattern in the medial and lateral hamstrings of OA patients was different from that of control subjects [30, 31]. From a biomechanical viewpoint, an increase in the adduction angle and moment in the frontal plane may lead to the muscle activity changes we observed in OA patients [15–17].

Peak knee compressive force in the knee joint during walking was 2.55 ± 0.30 BW (mean ± standard deviation) in the OA group, which is consistent with the previously reported results of 3.67 to 4.45 BW [32] and 1.6 to 1.8 BW [20] by considering the variety of subjects used in the different studies. The ML and AP components of knee joint force were firstly investigated in this study because OA in the knee joint is more sensitive to joint functional alterations in the frontal plane than in the sagittal plane [15–19]. In contrast to the AP component of the joint force, the ML component increased with OA. Because the direction of the ML component from medial to lateral corresponds to adduction moment in the knee, this result could explain why the adduction moment was higher in OA patients than is asymptomatic subjects [16–18].

The results indicated that EFAR and EFTR were higher in OA patients than in control subjects. Several previous studies reported 1.5–2.3 folds higher antagonistic cocontractions in OA patients than in normal subjects based on EMG measurements [3, 5, 9]. The muscle forces and torques generated by muscles in OA patients were similar to those in the asymptomatic group for flexor muscles, but much higher for extensor muscles. Given that the EFAR and EFTR indicate the level of cocontraction between the flexor and extensor muscle groups, the increase in both ratios is consistent with the experimental results. Because a biomechanical parameter has not been defined to reflect the level of co-contraction between several muscle groups the knee joint, for example, the quadriceps and hamstrings, the EFAR and EFTR may be used as indices of co-contraction. Significant differences in the EFAR and EFTR between OA patients and asymptomatic subjects indicate that these ratios can be applied to diagnose OA patients and analyze their kinematic and dynamic patterns.

There are several limitations and restrictions to this study. Anatomical information such as mass, center of gravity, moment of inertia, muscle attachment points, PCSA of the muscle, and maximum muscle forces was obtained from the literature and scaled based on the weight and height of subjects, because subject-specific information is difficult to obtain. In addition, only one type of objective function was utilized in this study to compute muscle forces. Parametric studies to determine the sensitivity of the model to anatomical information as well as the optimization scheme used would enhance the reliability of the analysis.

## 5. Conclusions

Inverse dynamic analysis and an optimization technique were applied to a lower extremity model with motion capture data to compare biomechanical parameters between OA patients and normal healthy subjects. OA patients had higher extensor muscle forces and lateral component of the knee joint force than normal subjects. Force and torque ratios of extensor and flexor muscles (EFAR and EFTR), which indicate the level of antagonistic cocontraction, were substantially higher in OA patients than in normal subjects. The presented findings and technologies provide insight into biomechanical changes in OA patients and can also be used to evaluate the postoperative functional outcomes of the OA treatments.

## Figures and Tables

**Table 1:** The mean ± standard deviation and *P* values of independent sample *t*-test between OA and asymptomatic groups in mean muscle forces and peak 3D knee joint forces as well as mean knee extensor/flexor muscle forces, torques, and ratios (EFAR and EFTR), where the muscle forces and the joint forces were normalized to the maximum isometric muscle forces and the body weight (BW), respectively.

	OA (*n* = 11)	Asymptomatic (*n* = 10)	*P* value
Mean muscle forces			
VINT	0.051 ± 0.020	0.020 ± 0.005	0.001*
VLAT	0.140 ± 0.070	0.057 ± 0.016	0.002*
VMED	0.130 ± 0.065	0.051 ± 0.014	0.002*
RF	0.220 ± 0.110	0.100 ± 0.047	0.006*
SM	0.027 ± 0.016	0.020 ± 0.010	0.230
ST	0.022 ± 0.012	0.015 ± 0.005	0.095
BFLH	0.043 ± 0.012	0.020 ± 0.008	<0.001*
BFSH	0.016 ± 0.004	0.025 ± 0.011	0.033*
GASM	0.088 ± 0.022	0.110 ± 0.025	0.053
GASL	0.064 ± 0.010	0.072 ± 0.019	0.300
SAR	0.028 ± 0.014	0.038 ± 0.015	0.170
GRA	0.069 ± 0.026	0.039 ± 0.017	0.006*
Peak 3D knee joint forces (BW)			
Compressive force	2.72 ± 0.21	2.47 ± 0.21	0.047*
ML shear force	0.43 ± 0.03	0.10 ± 0.01	0.001*
AP shear force	0.55 ± 0.12	0.70 ± 0.04	0.140
Mean extensor/flexor values			
Flexor activation	0.05 ± 0.02	0.05 ± 0.01	0.250
Flexor torque	0.22 ± 0.07	0.28 ± 0.03	0.057
Extensor activation	0.13 ± 0.05	0.05 ± 0.01	0.026*
Extensor torque	0.25 ± 0.08	0.19 ± 0.04	0.043*
EFAR	3.67 ± 1.14	2.00 ± 0.63	0.040*
EFTR	1.82 ± 0.40	1.27 ± 0.34	0.048*

*Significant differences between the OA patients and the asymptomatic group (*P* < 0.05).
